# Regulating appetite in broilers for improving body and muscle development – A review

**DOI:** 10.1111/jpn.13407

**Published:** 2020-06-26

**Authors:** Marinus F. W. te Pas, Randy Borg, Nico J. H. Buddiger, Benjamin J. Wood, Johanna M. J. Rebel, Marinus M. van Krimpen, Mario P. L. Calus, Jong‐Eun Park, Dirkjan Schokker

**Affiliations:** ^1^ Wageningen University and Research Wageningen Livestock Research Wageningen The Netherlands; ^2^ Cobb Vantress Boxmeer The Netherlands; ^3^ Hendrix Genetics North America Office Kitchener ON Canada; ^4^ Animal Genomics & Bioinformatics Division National Institute of Animal Science Rural Development Administration Jeonju Korea; ^5^ Present address: School of Veterinary Science University of Queensland Gatton QLD Australia

**Keywords:** biological mechanism, broiler, regulation, understanding and optimization of appetite

## Abstract

Appetite is the desire for feed and water and the voluntary intake of feed and is an important regulator of livestock productivity and animal health. Economic traits such as growth rate and muscle development (meat deposition) in broilers are directly correlated to appetite. Factors that may influence appetite include environmental factors, such as stress and temperature variation, and animal‐specific factors, such as learning period, eating capacity and preferences. Feed preferences have been reported to be determined in early life, and this period is important in broilers due to their fast growth and relatively short growth trajectories. This may be of importance when contemplating the use of more circular and sustainable feeds and the optimization of appetite for these feeds. The objective of this review was to review the biological mechanisms underlying appetite using data from human, animal and bird models and to consider the option for modulating appetite particularly as it relates to broiler chickens.

## 1 INTRODUCTION

The growing world population induces a growing demand for animal‐derived protein. While pork and beef have religious limitations in parts of the world, chicken meat is acceptable to many religions. In the past decades, broiler breeding improved the growth rate and feed efficiency, likely changing appetite in that process, thereby reducing the global footprint per unit of protein produced enormously, although this is related to health and welfare problems. Therefore, chickens are highly efficient producers of protein. Appetite is strongly related to environmental temperature and (subclinical) illness. Therefore, it may be expected that climate change will impact appetite, and thereby animal welfare, health and productivity (Patrick et al., 2004). As a consequence, the immune system of the animal may be affected by inducing the chronic low‐grade inflammatory state. But still, our understanding of the biological mechanisms describing the relationship between the animal's reaction to environmental temperature and appetite remains poor. Interestingly, experiencing increased temperatures during incubation seems to improve the ability of the animal to cope with increased temperatures later in life (Loyau et al., 2016). This suggests that epigenetic mechanisms are in place to cope with (gradually) increasing temperatures. Taken together, this shows that the growing demand of the future world population requires additional knowledge to further increase both animal welfare and productivity for future management, diets and genetics.

Appetite is the desire for feed and water (Blundell, 2010) and is important for all livestock animals. It is normally described in terms of hunger, satiation and satiety (Corfe, Harden, Bull, & Garaiova, 2015) and determines the voluntary feed intake of animals (Forbes, 1995, chapter 1). Feed provides nutrients for maintenance, growth and activity of the animal. This animal is then used for animal‐derived food products, mostly protein, for human consumption. In a world with a growing population, the demand for animal‐derived proteins is also growing. Added to this growth are also increased consumer wealth, particularly in developing economies, and consequently higher meat consumption per capita together with added population size (FAO, 2009).

A key component of a higher livestock productivity level is an appetite that supports that productivity. Several traits underlie the general description of livestock productivity. First, animal productivity may differ. Furthermore, depending upon the animal product the production trait may differ. This is usually defined for meat‐producing animals in terms of growth and protein deposition, or for dairy in terms of milk yield and milk protein and fat and for laying chickens in terms of reproduction, egg production and egg composition. Second, the efficiency with which these products are produced is usually in terms of amounts of feed consumed. Third, the health and resilience of the animal, animal welfare aspects, and lastly the appetite of the animal for specific feed are specific traits (Morrissey et al., 2014).

Animal production traits (e.g. meat deposition, reproduction, lactation and egg production) themselves are complex traits (te Pas, Madsen, Calus, & Smits, 2017) that are regulated by a combination of the genotype of the animal and the environmental influences on the animal and the interactions between the environment and the genotype (Cookson, Liang, Abecasis, Moffatt, & Lathrop, 2009; Dermitzakis, 2012; Kim, Larsen, Short, Plastow, & Rothschild, 2000). Breeding livestock animals for improved production and feed efficiency has been likely to have indirectly changed the appetite of these populations through correlated responses resulting from those selection goals. As such, decades of genetic selection for production traits may have considerably changed appetite in livestock populations. Examples include Siegel and Wisman (1966) showing an association between appetite and selection for body weight; Rubin et al. (2010) reporting loci related to growth, appetite and metabolic regulation; and Ahsan et al. (2013) who also found loci related to growth and appetite in chickens. However, possible dietary changes forced by climate change, or due to altered feed conditions, or competition between feed and food with the growing human population, may present a greater challenge for regulating appetite in the future (Hoffmann, 2010; Rosenzweig & Hillel, 1998). It will be important to understand the regulation of appetite under the present conditions to deal with possible future challenges. By utilizing breeding or feed composition changes, it may be possible to modulate appetite for challenging future environments.

## 2 OBJECTIVE

The objective of this paper is to review the regulation of appetite, including the role of the gut microbiome, with special focus on broiler chickens. We can foresee a time when competition for resources between humans and animals or changes in ability to supply the current feed for animals require alternatives to be identified without increasing the environmental footprint. Based on that knowledge, we discuss the options for modulating, modifying, changing and adapting appetite in a changing world dominated by a growing demand for animal‐derived protein food. In addition, physiological stress due to environmental (heat) stress will be discussed.

## 3 PHYSIOLOGY OF APPETITE

### 3.1 The importance of appetite

Without an appetite, there would be no feed intake. Feed provides the animal with nutrients for body development and maintenance, and energy for activity, among them activities to find and consume feed. Picard, Plouzeau, and Faure (1999) indicated the importance of energy for feed consumption in that eating feed is the major non‐resting activity of broilers. Searching for feed is also an important activity for animals living in a natural environment, but in the modern livestock management systems, there is ad libitum availability of feed. The activities to find feed are almost zero, so voluntary feed intake or willingness to eat (Forbes, 1995—chapter 1) is a important trait and appetite is a major component. Feed intake is easily measured (both quantitative and objective), but willingness (i.e. motivation) is more difficult to determine.

While total feed intake regulated by appetite is an important factor, the composition of the feed, feed conversion and nutrient absorption by the gut are also important for appetite. The willingness of the animal to eat, the appetite, is the major factor regulating nutrient uptake. While hunger may be a logical reason for eating feed, an appreciation that appetite can be regulated by other factors is also important. Such factors can be diverse physiological, neurological and environmental mechanisms (Acar, Patterson, & Barbato, 2001; Hassan, Elzubeir, & El Tinay, 2003; Lobaugh, Joshua, & Mueller, 1981; Morrissey et al., 2014; Portella, Caston, & Leeson, 1988; Siegel & Wisman, 1966).

## 4 FUTURE ENVIRONMENTAL CHANGES AND ITS POSSIBLE EFFECTS ON APPETITE

### 4.1 Climate change

Environmental variation induces physiological stress, which can lead to reductions in appetite. This in turn affects muscle development, health, immune system and other complex traits (Aldwin, 2007). Effects on the immune capacity may also make animals less resilient and vulnerable to disease, further reducing appetite and productivity (Lara & Rostagno, 2013; Lei, et al., 2013). Knowing this, climate change poses a threat to animal protein production due to increases in environmental variation. The costs for climate control of barns could rise, and where climate control is suboptimal, broiler appetite could be reduced (McMichael, Powles, Butler, & Uauy, 2007; Pelletier & Tyedmers, 2010; Weindl et al., 2015). Many questions can only be partly answered currently, such as “what is the effect of climate change on animal appetite?” and “what does this reaction do with animal productivity and more specifically, animal appetite?”. A better understanding of what appetite is and how it is regulated in the animal may add to our understanding of the answers to these questions.

### 4.2 Gut microbiome

The microbiome influences the uptake of nutrients from the feed, and it can also influence the behaviour of the animal in what it wants to eat (Corfe et al., 2015). Increases in environmental variation envisaged above may affect the gut microbiome composition. The microbiome is not only part of the digestive system, but it also synthesizes new nutrients that are absorbed by the animal and have specific functions including appetite regulation. Differential composition of the gut microbiome can make varying amounts of these nutrients, thereby differentially regulating traits including appetite. However, the composition of the feed also affects the composition of the gut microbiome (Corfe et al., 2015; Spor, Koren, & Ley, 2011). Consequently, balancing the relationship between environmental and dietary change will have an effect on appetite. By administrating the probiotic *Butyricicoccus pullicaecorum,* the feed conversion of the broilers was reduced as well as increased protection against potential harmful bacteria (Eeckhaut et al., 2008).

The genetic background of the host also plays a role in the establishment of the gut microbiome (Spor et al., 2011). This has been shown by Schokker et al. (2015) where two genetically divergent lines showed a different small intestinal microbiota composition in early life. Their results suggest that the genetically divergent lines have different coping mechanisms in early life regarding potential pathogenic threats. Additionally, other studies have shown the link between faecal microbiota and feed efficiency (Singh et al., 2014; Stanley et al., 2012). This shows that both feed (additives) and genetics may influence the immune competence and feed efficiency of birds, where feed efficiency is linked to appetite.

### 4.3 Genetic regulation of appetite versus environmental factors

The genotype of the animal plays a role in appetite as genes encoding for key regulatory factors such as hormones, neuropeptides, receptors, enzymes, transcription factors and binding/transport proteins constitute the molecular basis for regulatory systems. Be that sensing, signalling and metabolic pathways or the integration of the three. However, we do not yet have a complete understanding of the genetic basis for these regulatory pathways in poultry (Richards, 2003). In humans, there is clear evidence for genetic regulation of appetite‐related diseases such as anorexia nervosa (Bulik, van Slof‐Op't Landt, Furth, & Sullivan, 2007). Although the environment clearly had an impact, genome‐wide association studies (GWAS) and other studies showed evidence of a genetic effect on anorexia nervosa (Duncan, et al., 2017; Poyastro Pinheiro, Root, & Bulik, 2009). Conversely, overeating and obesity, in combination with the gut microbiome, have also been shown to be genetically controlled (Van der Klaauw & Sadaf Farooqi, 2015; Wang, Wang, Donovan, & Teran‐Garcia, 2013). Even the preferential appetite for specific nutritional components is also partly under genetic control (Bachmanov et al., 2016).

Genetic lines of broilers differ in the amount of feed consumed (Dr. Randy Borg PhD, Cobb‐Vantress, pers. comm.) and this indicates a genetic regulation of appetite in broilers, also reviewed in Richards (2003). This may be related to the selection for growth that has been a central breeding objective for many decades (Buzala & Janicki, 2016). Similar to humans, the molecular basis underlying this may be related to the regulation of the genes in the appetite‐controlling brain centres (Fang et al., 2014). Since the performance of the same broiler line reared in the United States and the EU differs, there must be an important genotype‐by‐environment (GxE) interaction. If the genotype is the same, the difference must be found in the environment, consisting mainly of management conditions and feed composition and its interaction with the genotype. There are significant broiler diet differences between North America and Europe with corn–soybean versus wheat‐based diets, respectively, contributing to the GxE interaction seen (Wood & Willems, 2014). Importantly, it should be noted that the interactions among feed composition, environment and genotype may affect the epigenome, which is important for the regulation of gene expression (Yadav & Maurya, 2014). Since feed composition directly affects the gut microbiome composition, which in turn influences the epigenome and animal metabolism, there is likely also an interaction between feed composition and gut microbiome that may affect appetite.

By understanding the changes in breeding programs and the associated feeding and management strategies (i.e. USA versus EU; including newly formulated future feeds), the regulating factors for appetite may be identified. It has been shown that in birds, feed conversion ratio has a low to moderate heritability (Sell‐Kubiak, Wimmers, Reyer, & Szwaczkowski, 2017). Additionally, as discussed earlier genetics and feed have an effect on appetite of the broilers. Thus, by typing the (faecal) microbiome of birds, it is possible to discriminate animals for appetite; this could be within a genetic line or between genetic lines. It is important to note that other factors including stress level, subclinical or chronic illness could affect this parameter too.

### 4.4 Regulation of appetite—palatability, appetite, hunger, satiation and satiety

The palatability of feed depends on the sensory properties, nutritional value, perception and appraisal of the animal. It is the combination of visual, smell, taste and texture properties that together constitute overall palatability. Chickens are able to detect odours, owing to a well‐developed olfactory system and the use of smell for searching for feed (Steiger, Fidler, Valcu, & Kempenaers, 2008). The nasal cavity and the right forebrain hemisphere seem to be important for olfactory recognition, memory and preferences, although the sense of smell in chickens is also sometimes questioned (Burne & Rogers, 2002; Jones & Roper, 1997; Steiger et al., 2008). Chickens learn to recognize specific olfactory cues related to feed during the last part of incubation and the early post‐hatching period (Burne & Rogers, 2002). After that period, memory and recognizing feeds are important in the willingness to eat (Barber & Kimbrough, 2015; Jones & Roper, 1997).

Chickens also have a well‐developed set of taste receptors for the different basic tastes (Erdoğan & Iwasaki, 2014; Ganchrow & Ganchrow, 1985, 1987). Kudo, Shiraishi, Nishimura, Bungo, and Tabata (2010) showed the relationship between the number of taste buds and sensitivity for a specific taste; this relationship may also affect feed preference. Interestingly, chicken breeds with higher weights have the highest average number of taste buds and higher taste detection sensitivity (Ganchrow & Ganchrow, 1987; Kudo et al., 2010). This may suggest that (a) the number of taste buds is genetically determined and possibly can be bred for, and (b) the number of taste buds is related to the appetite, as heavier chickens tend to eat larger meals. It may be argued that the number of taste buds is not specific to appetite, as larger animals will have larger organs and as a consequence will have more taste buds—and more taste buds may increase the feed appraisal leading to higher appetite. Alternatively, a higher appetite may require a higher number of taste buds, irrespective of organ or body size. However, data on causality of the relation between appetite and number of taste buds are still lacking.

The palatability of feed depends heavily on learned knowledge that a feed is safe and nutritious to consume. Especially for chickens, in nature this is learned during the first day post‐hatch (Barber & Kimbrough, 2015; Jones & Roper, 1997) and this is an important parameter of palatability. If fed with specific odorous feed products during this early post‐hatch period, a difference can be made to overall consumption in chickens.

Palatability is an important parameter inducing appetite, and appetite is described in terms of hunger, satiation and satiety. First, hunger is the physical feeling that the body needs feed, and it is the most important driver for appetite and eating. However, hunger is not directly measurable but needs to be inferred from objective physical conditions (Blundell et al., 2010). During eating, the point of satiation determines the meal size by terminating eating (Corfe et al., 2015). Satiety is the physiological state best described as “fullness,” probably best related to the amount of feed eaten and the awareness of it. Further eating is inhibited until the time when hunger provides the drive for the next eating period. Thus, satiation is the intra‐meal satiety, while satiety is post‐ingestion satiety or inter‐meal satiety.

## 5 PARAMETERS DETERMINING APPETITE

A number of physical and physiological parameters determine the satiation and satiety of animals, which regulate appetite. Parameters increasing and decreasing appetite are called orexigenic and anorexigenic parameters respectively.

### 5.1 Appetite control: orexigenic regulation

**FIGURE 1:**
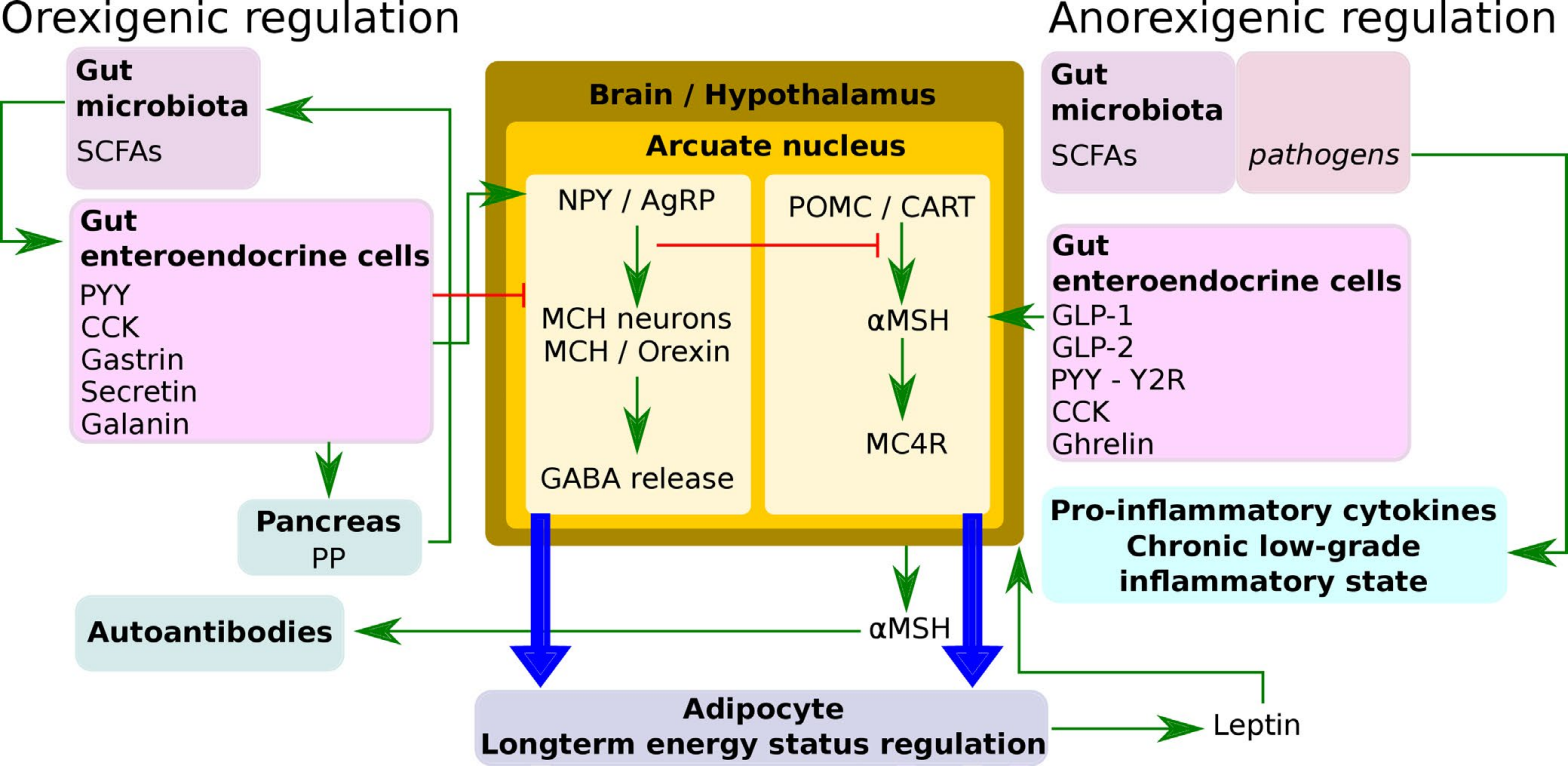
Orexigenic and anorexigenic regulation of appetite in broilers. The arcuate nucleus of the hypothalamus takes a central position in the regulation containing the orexigenic NPY/AgRP and the anorexigenic POMC/CART neurons. Peripheral influence of both areas in the arcuate nucleus is indicated. CCK, cholecystokinin; GABA, gamma‐aminobutyric acid; GLP, glucagon‐like peptide; MC4R, melanocortin‐4 receptor; MCH, melanin‐concentrating hormone; NPY/AgRP, neuropeptide Y/agouti‐related protein; POMC/CART, pro‐opiomelanocortin/cocaine‐ and amphetamine‐regulated transcript; PP, pancreatic polypeptide; PYY, peptide YY; SCFAs, short‐chain fatty acids; Y2R, neuropeptide Y receptor Y2; α‐MSH, alpha‐melanocyte‐stimulating hormone

Orexigenic appetite control increased feed intake and is shown in Figure 1. The orexigenic stimulus is regulated by both central and peripheral factors. Within the brain, the hypothalamic feeding circuits integrate the peripheral signals about nutritional status, and within the hypothalamus, the *arcuate nucleus* and the *lateral hypothalamic area* are particularly involved in appetite regulation. The *arcuate nucleus* consists of various regions sensitive to regulation by peripheral signals and secreting neuropeptides in response to these peripheral signals (Becskei et al., 2008; Sohn, 2015; Song, Everaert, Wang, Decuypere, & Buyse, 2013). For orexigenic appetite control, the neuropeptide Y and agouti‐related protein (NPY/AgRP) neurons and the pro‐opiomelanocortin, cocaine‐ and amphetamine‐regulated transcript (POMC/CART) neurons are important. Both are so‐called first‐order neurons (Argente‐Arizón, Freire‐Regatillo, Argente, & Chowen, 2015). The NPY and AgRP neurons are orexigenic neurons that have powerful local inhibitory synaptic connections with the anorexigenic POMC and CART neurons (indicating a strong neuronal circuit to influence appetite; Song et al., 2013). The NPY and AgRP neurons release the inhibitory neurotransmitter gamma‐aminobutyric acid (GABA) (Sternson & Atasoy, 2014), which mediates most of the orexigenic effects through inhibition of multiple anorexigenic neurons. Thus, the orexigenic effects are also dependent upon down‐regulation of the anorexigenic response. Second‐order orexigenic neurons release melanin‐concentrating hormone (MCH) and orexins (Sohn, 2015). However, the activity of orexins in chickens is uncertain since they did not stimulate feed intake in chickens (Richards, 2003). Both NPY and AgRP are also involved in the long‐term energy regulation storage in adipose tissue (Richards, 2003).

The *hypothalamus* is influenced by hormonal factors that cross the blood–brain barrier and non‐hormonal signalling via influencing neuronal circuits that bring the signal to the hypothalamus. An important system is the *microbiota–gut–brain axis*. This axis shows that bacterial‐derived factors can regulate brain function, while the brain can influence the microbiome via gastrointestinal motility, secretion and permeability, and also via signalling molecules released into the gut lumen by neurons (Carabotti, Scirocco, Maselli, & Severia, 2015). The gut microbiome affects the synthesis and secretion of gastrointestinal neuroendocrine hormones and signalling neuropeptides (Mu, Yang, & Zhu, 2016; Norris, Molina, & Gewirtz, 2013) via acting on specific enteroendocrine cells (Mu et al., 2016; Spreckley & Murphy, 2015). Among them are the orexigenic peptide YY (PYY), which is mainly synthesized in the small intestine, and signals to the brain either via crossing the blood–brain barrier or via stimulating the vagal efferent nerves (Psichas, Reimann, & Gribble, 2015; Rasoamanana, Darcel, Fromentin, & Tomé, 2012). It has been shown that the intestinal PYY mRNA expression changes significantly in response to the nutritional status of the chicken. Major metabolites synthesized by the gut microbiome are short‐chain fatty acids (SCFAs). SCFAs are detected by enteroendocrine cells via an array of specific receptors. Differential gut microbiome compositions can produce different SCFAs and express them at different levels, which may provide different signals. Colonic SCFA infusion increased the plasma PYY concentration considerably, and subsequently, PYY orexigenic effects may be induced.

Galanin is an orexigenic factor that is most abundant in the duodenum with lower concentrations in the stomach, small intestine and colon. (Kaplan, Spindeltt, Isselbacher, & Chin, 1988). The main target for galanin is the *hypothalamus*, although its effects are widespread in the brainstem and cortex (Gundlach, Burazin, & Larm, 2001). It has been shown that a *hypothalamic–brainstem integration network* allows homeostatic control of feeding (Rasoamanana et al., 2012). Another interesting mechanism involving the microbiome is appetite control via auto‐antibodies. Fragments of identical amino acid sequences shared by regulatory peptides and microbial proteins induce the synthesis of auto‐antibodies that influence the functions of the regulatory peptides (Fetissov et al., 2008). One example includes auto‐antibodies against alpha‐melanocyte‐stimulating hormone (α‐MSH) and mimicked by the bacterial caseinolytic peptidase B (ClpB) protein (Tennoune et al., 2014). This energy‐balanced regulator reduces feed intake and has an anorexigenic function. If auto‐antibodies reduce the functionality of this protein, the net results will be orexigenic. Tennoune et al. (2015) reported that supplementing rats with *E. coli* K12 changed levels and affinity of α‐MSH‐reactive IgG (but not IgM) in female rats, which were increased and associated with positive energy balance. In contrast, α‐MSH immunoglobulin (Ig)M (but not IgG) levels in male rats increased and were associated with α‐MSH‐mediated satiety and anxiety. According to these authors, the reason behind the sex differences in α‐MSH auto‐antibody production might be related to the presence of *E. coli* in the resident microbiota before *E. coli* gastrointestinal supplementation in females but not in males. In chickens, the effect of α‐MSH on appetite has been shown (Cline et al., 2008; Richards, 2003), but to the best of our knowledge, auto‐antibodies for α‐MSH have not been reported, although auto‐antibody synthesis has been shown in chickens (Albini, Wick, Rose, & Orlans, 1974).

The orexigenic peptide pancreatic polypeptide (PP) released from PP cells in the pancreas showed increased expression after fasting. The excitation of the vagus nerve and the administration of gastrin, secretin or cholecystokinin induce PP secretion. PP regulates various pancreas functions such as self‐regulation of pancreatic secretion activities (endocrine and exocrine). PP also affects hepatic glycogen levels and gastrointestinal secretions (Richards, 2003).

### 5.2 Appetite control: anorexigenic regulation

Anorexigenic appetite control decreases feed intake regulated by both central neural and peripheral factors and is shown in Figure 1. Similar to orexigenic appetite control, the *arcuate nucleus* of the *hypothalamus* integrates the peripheral signals about nutritional status and controls appetite. For anorexigenic appetite control, the first‐order POMC and CART neurons are important. Multiple second‐order anorexigenic neurons receive GABAergic input from the NPY and AgRP neurons (Sternson & Atasoy, 2014). The POMC and CART neurons suppress feed intake by releasing α‐MSH, which functions as an agonist of the anorexigenic melanocortin receptors, primarily MC4‐R, both centrally and peripherally (Sohn, 2015). Long‐term energy regulation storage in adipose tissue is mainly regulated via α‐MSH and the adipose‐secreted leptin (Richards, 2003; Sohn, 2015).

The *microbiota–gut–brain axis* regulates anorexigenic appetite control via several independent different mechanisms. The enteroendocrine L cells of the small intestine secrete the anorexigenic hormones glucagon‐like peptide 1 (GLP‐1) and glucagon‐like peptide 2 (GLP‐2) (Rasoamanana et al., 2012; Richards, 2003; Ripken, 2016). They were found to co‐localize in the same secretory granules (Monir, Hiramatsu, Nishimura, Takemoto, & Watanabe, 2014; Nishimura, Hiramatsu, Monir, Takemoto, & Watanabe, 2013). The transcripts for the GLP‐1 and GLP‐2 receptors are expressed through the entire gastrointestinal tract (Honda, 2016). Therefore, it has been suggested that GLP‐1 and GLP‐2 signal to the brainstem via the vagal afferent nerves (Honda, 2016). Presently, it remains unknown whether the brain contains GLP receptors, and the regulation of the expression of the GLPs related to a meal is poorly understood. It is important to note that the anorexigenic effects in chickens are functional at very low expression levels of the GLP‐1 and GLP‐2, suggesting that these proteins may play a prominent role in regulating feed intake (Honda, 2016). Furthermore, the GLPs are also synthesized by the pancreas and the brain (Honda, 2016). Apart from the above‐described orexigenic effects of SCFAs, there is evidence for SCFA‐induced increased GLP‐1 levels in enteroendocrine cells and blood (Wichmann et al., 2013; Yadav, Lee, Lloyd, Walte, & Rane, 2013), thereby inducing anorexigenic effects. This suggests that the gut microbiome composition may regulate feed intake in two directions.

The PYY anorexigenic hormone is also highly expressed in the chickens’ small intestine, mainly the jejunum (Ripken, 2016). In chickens, it binds preferentially to the neuropeptide Y receptor Y2 (Y2R) (Aoki et al., 2017). The Y2R gene is expressed in the appetite‐regulating brain centres, suggesting that PYY can cross the blood–brain barrier (Aoki et al., 2017). However, PYY also signals via the vagal afferent nerves (Aoki et al., 2017).

In particular, when fat and protein in the feed enter the stomach, the chickens’ duodenum enteroendocrine cells release cholecystokinin (CCK) (Psichas et al., 2015; Rasoamanana et al., 2012). In chickens, CCK stimulates satiation and thus limits meal size (Ripken, 2016). The central CCK receptor (CCKAR) mediates the CCK effect in the brain, while peripheral CCK effect is mediated via the peripheral CCK receptor (CCKBR) (Rodríguez‐Sinovas, Fernández, Manteca, Fernández, & Goñalons, 1997). Importantly, chickens with a high genetic potential for growth, such as broiler‐type chickens, are relatively resistant to the anorexigenic effects of exogenously administered CCK, suggesting that the satiety set point in these chickens has been altered (Dunn et al., 2013). These chickens show low expression of the CCKAR gene in the brain (Dunn et al., 2013). This was reported to correlate with increased levels of the orexigenic AgRP in the hypothalamus (Dunn et al., 2013). Other important gastrointestinal anorexigenic peptides include serotonin (Rasoamanana et al., 2012; Richards, 2003), gastrin that stimulates gastric acid synthesis, and bombesin, or gastrin‐releasing peptide (Furuse, 1999; Richards, 2003). Differing to mammals, in chickens, the ghrelin peptide hormone has an anorexigenic function (Geelissen et al., 2006; Kaiya, Kangawa, & Miyazato, 2013; Kaiya, Miyazato, Kangawa, Peter, & Unniappan, 2008; Ocłoń & Pietras, 2011; Richards, 2003; Richards & McMurtry, 2010; Saito et al., 2002; Seim, Jeffery, Herington, & Chopin, 2015). The hormone is widely expressed in many tissues including brain (the hypothalamus), duodenum, pancreas, spleen and liver, but highest expression is in the proventriculus or the glandular part of the avian stomach (Buyse, et al., 2009). The proventriculus and plasma ghrelin concentration increase gradually with age, and the plasma concentration is positively correlated with the proventriculus concentration, suggesting that plasma ghrelin has been synthesized in the proventriculus (Kitazawa, Hiraga, Teraoka, Yaosaka, & Kaiya, 2015; Wada, et al., 2003). After a 12‐hr fasting, the ghrelin concentration was increased and only decreased after six hours of eating (Kaiya et al., 2008). This suggests that ghrelin is a hunger signal like in mammals, but contrary to mammals, ghrelin inhibits feed intake (Geelissen et al., 2006; Kaiya et al., 2008, 2013; Ocłoń & Pietras, 2011; Richards, 2003; Richards & McMurtry, 2010; Saito et al., 2002; Seim et al., 2015). This suggests that different pathways are operating in mammals and birds. In mammals, ghrelin acts on the central orexigenic NPY–orexin system (ghrelin is able to cross the blood–brain barrier), while ghrelin acts in birds on the anorexigenic central corticotropin‐releasing factor (CRF) system (Kaiya et al., 2013). The exact biological mechanisms are unclear, and several mechanisms have been proposed (Kaiya et al., 2013). For example, while CRF is itself a strong inhibitor of feed intake, it has been suggested that in birds, the effect is mediated by a CRF family member, urocortin (Khan, Kaiya, & Tachibana, 2014; Ogino, Okumura, Khan, Cline, & Tachibana, 2014). In chickens, no interaction between ghrelin and the NPY neurons was observed (Saito et al., 2005). A potential mechanism is that ghrelin stimulates the central activity of CRF, thereby activating the *hypothalamus–pituitary–adrenal axis* to increase the adrenal release of corticosterone. Nevertheless, ghrelin remains a hunger‐induced hormone that reduces feed intake, thereby worsening the hunger. This suggests a strong positive feedback loop that somehow has to be broken because, if unbroken, the animal will die due to starvation. An altered appetite is required to maintain productivity.

Feed intake relates to health and well‐being of the animal. Although disease itself is outside the objectives of this review, stress induced by changed feed composition and thermal stress will be discussed. Stress is a major anorexigenic stimulus (Groesz et al., 2012; Matteri, Carroll, & Dyer, 2000). Animals that are out of homeostasis eat less than animals in homeostasis. It should be emphasized that even when the animals are not yet clinically ill, subclinical illness can induce the same physiological reactions, although often at a lower level (te Pas, Kruijt, Koopmans, & Smits, 2013). The issue of subclinical illness is that no signs of illness can be observed while there is a physiological response, which is often unrecognized and not correctly understood. Sickness can easily be induced by administration of pro‐inflammatory cytokines or cytokine inducers, for example by bacterial endotoxins such as lipopolysaccharides (LPS). Gregory, Payne, Devine, and Cook (2009) showed that administering LPS induced similar sickness behaviour in mammals and in chickens. It has been observed that pro‐inflammatory cytokines can promote catabolism via direct effects on several tissues including skeletal muscle and adipose tissue (Johnson, 1997). Cytokine signalling targets the central nervous system at specific sites. In the *hypothalamus,* both the NPY and AgRP and the POMC and CART neurons are downstream targets of cytokines, especially leading to loss of appetite due to peripheral inflammation (Krasnow & Marks, 2010). A second mechanism includes a chronic low‐grade inflammatory state characterized by overexpression of circulating inflammatory factors (Tan, Liu, Guo, Applegate, & Eicher, 2014). This status is associated with increased circulating levels of acute‐phase proteins and pro‐inflammatory cytokines (Bertoni, Trevisi, & Lombardelli, 2009) and has been associated with *stress* and *obesity* (Capuron & Miller, 2011). The adipose tissue expresses cytokines, which affects the brain to induce an anorexic response. The low‐grade inflammatory state reduces appetite (Johnson, 1997). It has been suggested that mild microbial challenges that do not result in clinical phenotypes may be the causal factor (Khadem, Soler, Everaert, & Niewold, 2014; Niewold, 2007; Soler et al., 2016).

## 6 APPETITE CONTROL: PERIPHERAL BIOLOGICAL MECHANISMS REGULATING APPETITE

Next to central regulation of appetite in the brain, other regulatory mechanisms outside the brain stimulating or inhibiting appetite are known. Such mechanisms act through regulating the metabolism. Known physiological mechanisms are visualized in pathways, which can be found in databases such as the Kyoto Encyclopedia of Genes and Genomes (KEGG) (https://www.genome.jp/kegg/; Kanehisa & Goto, 2000): The KEGG is a database resource for understanding high‐level functions and utilities of the biological system from large‐scale molecular‐level information. We will highlight pathways related to regulation of appetite. We will provide a direct Internet link to these pathways, where the visualization can be found and interactively searched for individual genes with specific gene information. It should be noted that feeding behaviour can be a measure of appetite: A genetic screen for feeding behaviour in pigs highlighted the genetic architecture and several pathways (Ding et al., 2018).

### 6.1 The AMPK pathway

The AMP‐activated protein kinase (AMPK) is a serine–threonine kinase. The AMPK system acts as a sensor of cellular energy status, activated by increases in the cellular AMP:ATP ratio (Hardie, Ross, & Hawley, 2012). This ratio is the result of metabolic processes either reducing the ATP production (e.g. deprivation of glucose due to feed restriction) or accelerating ATP consumption (e.g. activity). To activate AMPK, a threonine residue on its catalytic alpha‐subunit is phosphorylated by upstream kinases including liver kinase B1 (LKB1), calcium/calmodulin kinase kinase‐beta (CaMKK‐beta) and TGF‐beta‐activated kinase‐1 (TAK‐1). Activated AMPK reduces energy‐consuming biosynthetic pathways, such as protein, fatty acid and glycogen synthesis, and activates ATP‐producing catabolic pathways, such as fatty acid oxidation and glycolysis. The KEGG pathway shows a visualization of the AMPK pathway: https://www.kegg.jp/kegg‐bin/highlight_pathway?scale=1.0&map=map04152&keyword=ampk. The importance of the pathway is underlined by the fact that the pathway is connected to 19 other pathways, including the mTOR pathway. Indeed, it was shown that co‐expressed pathways including the AMPK pathway regulate many aspects of feed intake including minerals and other components (Da Silva Diniz et al., 2019).

### 6.2 The mTOR signalling pathway

The mammalian (or sometimes called: mechanistic) target of rapamycin (mTOR) pathway regulates homeostasis by directly influencing protein synthesis, transcription, autophagy, metabolism and organelle biogenesis and maintenance. mTOR contains two complexes, mTOR complex 1 (mTORC1) and mTOR complex 2 (mTORC2). mTOR is a kinase that links with other proteins and serves as a core component to form protein complexes. mTORC1 contains mTOR, Raptor, PRAS40, Deptor, mLST8, Tel2 and Tti1. mTORC1 is activated by growth factors, amino acids, energy status, stress and oxygen levels to regulate biological processes, including lipid metabolism, autophagy, protein synthesis and ribosome biogenesis. mTORC2, contains mTOR, mSin1, Rictor, Protor, Deptor, mLST8, Tel2 and Tti1, responds to growth factors and controls cytoskeletal organization, metabolism and survival. The mTOR complexes regulate cell growth, cell proliferation, cell motility, cell survival, protein synthesis, autophagy and transcription. mTORC2 promotes the activation of insulin receptors and insulin‐like growth factor 1 receptors which have been implicated in the control and maintenance of the actin cytoskeleton (Hay & Sonenberg, 2004; Jacinto et al., 2004; Lipton & Sahin, 2014; Yin et al., 2016). These data suggest that the many energy homeostasis functions of the AMPK pathway are regulated via the mTOR pathway. The mTOR pathway is visualized in the KEGG database: https://www.kegg.jp/kegg‐bin/highlight_pathway?scale=1.0&map=map04150&keyword=mtor. The mTOR pathway has numerous connections with other signalling pathways to regulate all its functions. The KEGG database mentions 55 links with other pathways.

### 6.3 The glucocorticoid–NPY relation: the NPY Adipocytokine signalling pathway

Appetite regulates feed intake. Feed intake regulates adipocyte volume and number, which in reverse regulates feed intake and probably also appetite—making this loop a crucial mechanism. Increased adipocyte volume and number are positively correlated with leptin production and negatively correlated with production of adiponectin (Meier & Gressner, 2004; Okamoto, Kihara, Funahashi, Matsuzawa, & Libby, 2006). Leptin is an important regulator of energy intake and metabolic rate primarily by acting at hypothalamic nuclei. Leptin exerts its anorectic effects by modulating the levels of neuropeptides such as NPY, AGRP and alpha‐MSH. Adiponectin lowers plasma glucose and FFAs. These effects are partly accounted for by adiponectin‐induced AMPK activation, which in turn stimulates skeletal muscle fatty acid oxidation and glucose uptake. Furthermore, activation of AMPK by adiponectin suppresses endogenous glucose production. The mTOR pathway relates to this pathway via interaction with the pro‐inflammatory cytokine TNF‐alpha implicated in regulation of insulin signalling, which is also important for regulation of feed intake. The KEGG database visualized the NPY Adipocytokine signalling pathway: https://www.kegg.jp/kegg‐bin/highlight_pathway?scale=1.0&map=map04920&keyword=npy. Glucocorticoids are tightly regulated, and the KEGG database visualizes a number of glucocorticoid receptor agonists and antagonists: https://www.kegg.jp/kegg‐bin/highlight_pathway?scale=1.0&map=map07225&keyword=glucocorticoid.

### 6.4 The Thyroid hormone signalling pathway

The thyroid hormones (THs) are important regulators of metabolism, which is related to feed intake and appetite, as discussed above (McAninch & Bianco, 2014). Thyroid hormones, L‐thyroxine (T4) and T3 (3,5,3'‐triiodo‐L‐thyronine), enter the cell through transporter proteins. T3, the active cellular form, binds to nuclear thyroid hormone receptors, which functions as a ligand‐dependent transcription factor and controls the expression of target genes. Thyroid hormone also acts via the integrin receptor, which has distinct binding sites for T3 and T4. One binding site binds only T3 and activates the phosphatidylinositol 3‐kinase (PI3K) pathway. The other binding site binds both T3 and T4 and activates the ERK1/2 MAP kinase pathway. The thyroid hormone pathway is directly linked with the mTOR pathway. The thyroid hormone pathway is visualized by the KEGG database: https://www.kegg.jp/kegg‐bin/highlight_pathway?scale=1.0&map=map04919&keyword=mtor.

### 6.5 The TGF‐β signalling pathway

A wide spectrum of cellular functions such as proliferation, apoptosis, differentiation and migration is regulated by TGF (transforming growth factor)‐beta family members including TGF‐βs, activins and bone morphogenetic proteins. While the TGF‐βs only bind to the type II receptor, recruitment and activation of the type I receptor to phosphorylate Smads are necessary for activity. In the nucleus, Smad complexes activate specific genes through cooperative interactions with other DNA‐binding and co‐activator (or co‐repressor) proteins. Alexandre et al. (2019) showed that the TGF‐β‐mediated mechanism is a key regulator of feed efficiency in cattle. The TGF‐β signalling pathway is visualized in the KEGG database: https://www.kegg.jp/kegg‐bin/highlight_pathway?scale=1.0&map=map04350&keyword=tgf.

## 7 STRESS INDUCED BY NOVEL FEED

Chickens learn what they can eat very early in life, during the last part of the incubation and the first day post‐hatch (Jones & Roper, 1997). One‐day‐old chicks showed graded responses to different concentrations of odours and demonstrate differential sensitivity to different odorants (Burne & Rogers, 1996). The role of olfaction on feed intake, as indicator for appetite in chickens, is still unclear. Jones and Roper (1997) showed that feed neophobia based on olfactory cues may become increasingly apparent if locally available and often odoriferous by‐products are added to the feed.

By 72–96 hr post‐hatch or possibly earlier, it becomes difficult to teach chickens to appreciate different feeds. Chicks apparently “learn” about their olfactory environment during the latter part of incubation and in the early post‐hatching period and the memory formed alters behaviour on the first day post‐hatch (Burne & Rogers, 1999). It is assumed that young chickens learn what is safe to eat and nutritious. Chickens may refuse to eat certain feeds or eat at a very low level reducing growth and potential productivity. The future may require chickens to eat different feeds than are currently used, and care should be taken to introduce the new feed composition during the correct phase in life.

## 8 ENVIRONMENTAL STRESS

Stress is the biological response of an animal to stimuli that disturb normal physiological homeostasis (Selye, 1976). Environmental stressors, particularly heat stress, are one of the most important challenges for livestock, including poultry production worldwide (Lara & Rostagno, 2013). The estimated annual economic costs of heat stress are $1.69–2.36 billion for the livestock industry, with $128–165 million for the poultry industry alone (Baumgard & Rhoads, 2013; St‐Pierre, Cobanov, & Schnitkey, 2003). For broilers, chronic heat stress has been reported to reduce feed intake by over 16% and body weight by over 32%, and increases the feed conversion ratio by over 25% at 42 days of age (Sohail et al., 2012). Adaptation to heat stress occurs, and gene expression is regulated throughout the day associated with daytime heat. Genes are regulated differently in adapted and non‐adapted chickens (te Pas et al., 2019, and unpublished results; Park et al., 2019; Srikanth et al., 2019). Furthermore, the heavier the birds, the higher the mortality risk (Drain, Whiting, Rasali, & D’Angiolo, 2007). Birds seem to be particularly sensitive to heat stress: due to a higher metabolic activity, modern poultry genotypes produce more body heat (Felver‐Gant, Mack, Dennis, Eicher, & Cheng, 2012; Lara & Rostagno, 2013; Mack, Felver‐Gant, Dennis, & Cheng, 2013; Soleimani, Zulkifli, Omar, & Raha, 2011). The stress these animals experience can not only affect their productivity, but it can also affect the composition of the product (i.e. meat and eggs) due to changed physiological mechanisms.

For humans, a confounding factor is that stress in livestock animals can have deleterious effects on food safety, for example, by increased vulnerability of the animals for pathogens such as *Salmonella* and *Campylobacter* (Jorgensen et al., 2011; Quinteiro‐Filho et al., 2010, 2012; Van der Fels‐Klerx, Jacobs‐Reitsma, Van Brakel, Van Der Voet, & Van Asselt, 2008; Wales, Breslin, Carter, Sayers, & Davies, 2007; Zdragas et al., 2012).

Finally, heat stress affects the *gut microbiome composition* and SCFA synthesis capacity; the importance for appetite has been discussed above (Tajima et al., 2007; Uyeno et al., 2010). Heat stress, and the more general stress, reduces feed intake by reducing eating time by reducing feeding‐related activity and increasing the time for drinking (Mack et al., 2013; Selye, 1976). Further behavioural adaptations include increased panting and spending more time for resting with elevated wings (Mack et al., 2013). The reduced appetite, feed intake and changed behaviour seriously affect the animals’ physiology, productivity and health. Panting increases blood carbon dioxide levels and pH, which hampers bicarbonate availability and decreases free calcium levels in the blood, reducing *eggshell quality* (Marder & Arad, 1989). Contrary, Calder and Schmidt Nielsen (1968) showed that the respiratory system of birds is a more effective gas exchange system than that of mammals although alkalosis during panting cannot be prevented. Heat stress activates the *hypothalamic–pituitary–adrenal axis* leading to elevated plasma corticosterone levels and decreased T3 levels—the latter may delay the onset of puberty (Elnagar, Scheideler, & Beck, 2010; Geraert, Padilha, & Guillaumin, 1996; Mack et al., 2013; Star, Decuypere, Parmentier, & Kemp, 2008; Yahav & Hurwitz, 1996). Another important aspect of heat stress is modulation of the immune response by the central nervous system (CNS) (Butts & Sternberg, 2008; Downing & Miyan, 2000; Padgett & Glaser, 2003). The immunosuppressing effect of heat stress is associated with reduced weights of thymus, spleen, lymphoid organs, bursa and liver (Bartlett & Smith, 2003; Felver‐Gant et al., 2012). Furthermore, reduced phagocytic ability of macrophages and altered levels of circulating cells together with an increased heterophil:lymphocyte ratio have been reported (Felver‐Gant et al., 2012; Prieto & Campo, 2010). As a consequence, reactive oxygen species (ROS) levels increase and the body enters the stage of oxidative stress starting to increase heat‐shock protein (HSP) synthesis aiming to activate cellular survival mechanisms.

Short‐term acute heat stress has been reported to be associated with increased ghrelin mRNA levels in the proventriculus, duodenum and the jejunum, and decreased CCK mRNA levels in the duodenum (Lei et al., 2013). An example of acute heat stress is circadian temperature cycles, especially in tropical countries, but the incidence in the rest of the world may increase due to climate change. Increased temperatures (from 37.8 to 39.5°C between embryonic days 7 and 16 for 12 hr per day) during incubation were shown to positively correlate with mortality rate of older chickens (32°C for 5 hr on day 34 post‐hatch) (Loyau et al., 2016) suggesting epigenetic modifications (Yossifoff, Kisliouk, & Meiri, 2008). It may be that the modification of temperature profiles in incubation could be used as a method to improve appetite and performance in changing environments. The production‐related effect needs to be investigated, but clearly modified gene expression patterns will have effects on production characteristics.

## 9 DISCUSSION: TO IMPROVE APPETITE

### 9.1 Knowledge requirements for improving appetite in broilers

All animal traits are regulated by biological mechanisms (Andersson, 2012; te Pas et al., 2017). Biological mechanisms include a number of cooperating genes acting in a pathway or network, which is influenced by environmental factors (te Pas et al., 2008; te Pas et al., 2017), implying that most of those traits are complex traits. In a recent review, we evaluated the existing knowledge about complex traits and how to investigate these traits (te Pas et al., 2017). For the complex trait, “balanced regulation of appetite” measurement and integration of specific data will be necessary. To mention some of these specific traits that directly relate to feed intake: (a) voluntary feed intake and related physical activity, (b) body temperature, (c) body development during life and (d) sudden death syndrome. In addition, health‐related traits including immunological and physiological traits and (sub)‐clinical physiology need to be measured. Environmental data, such as temperature, humidity and dust, and welfare traits, such as hunger and willingness to eat, can affect both these measurements of the animals’ physiology and should therefore be included. Here, it should be noted that location (e.g. USA versus EU) may create contrasting environments differently influencing animals from the same genotype. Finally, the microbiome composition and its metabolic capacity and activity should be measured. There may be a relation among appetite, microbiome composition and microbiome metabolic activity similar to the relations found in obesity. Together, the underlying biological mechanisms affect organ‐specific and body‐wide metabolism, which should ideally be measured too. All these phenotypes are the results of the interaction of the environment with the genome by influencing genome‐wide gene expression, affecting the (epi)genome and interacting with genomic variation. While this likely applies for many complex traits, the associated big data that will be generated may generate (new) specific challenges. Sensor technology can help with many of the measurements, and omics technology can add to the traditionally performed laboratory measurements.

With the enormous amount of data collected, the analysis requires a big data approach (Feltus et al., 2015; te Pas et al., 2017), but what will this knowledge bring us? The data will give insight into: (a) appetite measured as voluntary feed intake and physical activities to find feed or feeding associated with environmental conditions, including feed composition and temperature. It will give insight into what to expect for appetite in the future, as appetite will be affected by novel protein sources (e.g. used as feeds in the future) and by different levels of climate change. These insights are especially important if we proceed towards the concept of the circular economy (Geissdoerfer, Savaget, Bocken, & Hultink, 2017). (b) The relation between appetite for traditional and novel feed formulations, voluntary feed intake and the occurrence of sudden death syndrome. This will help to reduce sudden death syndrome while maintaining productivity through improved feeding. (c) The relations among appetite, body development, and related novel feed composition. (d) How the animal maintains metabolic homeostasis under different environmental conditions. This will help to further improve welfare and productivity of broiler lines. (e) The genomic regulation of appetite and how the animal regulates its genome in reaction to the environment will enable us to manage appetite. Above it has been argued that epigenetic regulation may be expected, and this will also have effect on the gene expression and on the activity of physiological pathways and networks, which also regulate metabolic activity body‐wide to maintain metabolic homeostasis. (f) Whether a specific gut microbiome composition is favourable—in combination with a specific feed in a specific environment in specific chicken lines—additional research could highlight the possibilities to replace the gut microbiome of all animals with the most favourable microbiome composition. (g) How breeding can further optimize the appetite, and what specific differences are required by different environmental conditions to come to optimal appetite for each environment.

Finally, it should be mentioned that if broilers eat more due to an increased feeling of hunger, they may search more aggressively for feed (a positive aggression), but they may also behave more aggressively, for example, via feather pecking (a negative aggression). When investigating options for increased appetite, the level of aggressiveness should be monitored as well (Savory, 1995). However, keep in mind that feather pecking could also result from feed or nutrient deficiencies, for example fibres or other substrates. For example, it is known that the risk of feather pecking is lower, and the time spent feeding is greater, with mash diets than with pelleted ones (Savory, 1995).

### 9.2 Optimizing broiler appetite

Meanwhile, what can we do now to improve knowledge and understanding of the appetite of broilers including dietary interventions without knowing all this? Appetite is about how to experience feed and voluntary feed intake, about palatability and feed composition. Thus, it would be interesting trying to influence appetite via the feed offered: “Variety is the spice of life,” indicating that new tastes could be a game‐changer. However, in chickens we have to focus on early life, that is the last part of the incubation period and the first day(s) post‐hatch, where (aversive) taste learning takes place (Burne & Rogers, 1997; Tiunova, Bezryadnov, & Anokhin, 2015). This does not mean that it is impossible to feed novel feeds to chickens, but especially for odorous novel feeds it may be advisable to use the early life learning period to establish a memory. So, it may take more than one generation to fully adapt the feeding habits of chickens. Nutritional attenuation of appetite can potentially be done via appetite‐stimulating biological mechanisms or via reducing appetite inhibitory mechanisms. An example of the first one is that chickens can learn to eat odorous feed, which they usually do not like. This enables the use of (by)products for feeding the animals. One example for this can be the leftovers of oil‐producing plants, like sunflower product for human consumption. Candidates for the second option are, for example, feed additives such as (a) essential amino acids or fats if the local feed is non‐optimal, (b) L‐arginine, an essential amino acid, which has been shown to reduce the LPS‐induced response in chickens (Tan et al., 2014) and suggested to reduce the low‐grade inflammatory state (Tan et al., 2014), (c) RRR‐α‐tocopherol succinate, a vitamin E analogue with special properties including immunomodulation by reducing cytokine production and cytoprotection and (d) phytobiotics, plant‐derived additives added to the feed, with anti‐inflammatory activities, for example curcumin, which has been shown to have anti‐inflammatory and antioxidant activities and an excellent safety profile. However, in a goldfish model intra‐peritoneal injection of curcumin reduced feed intake. Similar effects have been reported in humans (Aggarwal & Harikumar, 2009; Basnet & Skalko‐Basnet, 2011; Chainani‐Wu, 2003; Kang et al., 2011; Menon & Sudheer, 2007; Taillon & Andreasen, 2000; Zhang et al., 2015; https://turmericsgold.com/conditions/curcumin‐for‐weight‐loss/). The use of curcumin as feed additive may also improve the meat quality and antioxidant profile of breast muscle (Zhang et al., 2015).

Genetics can also change appetite in broilers on a larger scale. Feed intake is under genetic control in chickens (Barbato, 1994; Barbato, Siegel, & Cherry, 1982). Also, in general taste perception has been shown to be under genetic control (Bachmanov & Boughter, 2012). Furthermore, the number of taste buds correlates with appetite in broilers (Kudo et al., 2010). Although to the best of our knowledge no heritability is known for taste bud numbers, potentially it is possible to breed for higher number of taste buds that may regulate general appetite. Heavier breed chickens have more taste buds than lighter chicken breeds (Ganchrow & Ganchrow, 1987), suggesting that breeding to increase taste bud numbers may favour appetite and may adapt chickens to appreciate novel feeds. According to these authors, the number of taste buds varies between chicken within and between lines, but is stable within an animal after hatching and can therefore be measured early in life supporting selection of animals. Selection for specific taste buds can probably also change feed preference towards feeds related to a circular economy or towards diversification of the feed preferences. It is unclear whether breeding may change the adaptation period of feed preferences.

We also propose to investigate possibilities to select for anti‐anorexigenic mechanisms such as seen in broilers. For example, CCK, ghrelin and leptin effects may be changed to improve appetite. To do so, it requires quantitative knowledge of the relations among the expression levels of these hormones and growth rate, body size and muscle deposition (selection criteria). Such technical options may be long‐term sustainable solutions. It is important to note that the knowledge about nutritional attenuation in chickens is scarce, and using experiments in mammals (e.g. mice) may give unexpected results that may not relate to chickens. Therefore, this has to be performed with care. However, animals provided the feed (additives) and (changed) nutrition components could be used in the above‐mentioned research to verify the effects in chickens.

### 9.3 Recommendations

Since appetite is a complex trait, it is recommended to consider analysing changes at many different levels including genetics and in different environments keeping in mind the expected (future) environmental changes, including increased temperatures and novel (circular/sustainable) feeds. Furthermore, the ghrelin example shows that birds and mammals differ for mechanisms regulating appetite, and therefore, it may require experimentation with chickens instead of solely relying on mammalian‐related literature.

## ANIMAL WELFARE STATEMENT

The authors confirm that the ethical policies of the journal, as noted on the journal's author guidelines page, have been adhered to. No ethical approval was required as this is a review article with no original research data.
